# Dual-Antigen System Allows Elimination of False Positive Results in COVID-19 Serological Testing

**DOI:** 10.3390/diagnostics11010102

**Published:** 2021-01-11

**Authors:** Andrei Komarov, Anna Kaznadzey, Yue Li, Maria Kireeva, Ilya Mazo

**Affiliations:** 1 VirIntel, LLC, Gaithersburg, MD 20877, USA; andrey.komarov@virintel.com (A.K.); maria.kireeva@virintel.com (M.K.); Ilya.Mazo@virintel.com (I.M.); 2 Institute for Information Transmission Problems, RAS, Bolshoy Karetny per. 19, 127051 Moscow, Russia; 3 Health Informatics & Data Science Georgetown University, Georgetown University Medical Center, 4000 Reservoir Road, NW, Washington, DC 20007, USA; yl1168@georgetown.edu; 4 Argentys Informatics, LLC, Gaithersburg, MD 20877, USA

**Keywords:** COVID-19, SARS-CoV-2, serological test, ELISA, dual-antigen

## Abstract

Determining the presence of antibodies in serum is important for epidemiological studies, to be able to confirm whether a person has been infected, predicting risks of them getting sick and spreading the disease. During the ongoing pandemic of COVID-19, a positive serological test result can suggest if it is safe to return to work and re-engage in social activities. Despite a multitude of emerging tests, the quality of respective data often remains ambiguous, yielding a significant fraction of false positive results. The human organism produces polyclonal antibodies specific to multiple viral proteins, so testing simultaneously for multiple antibodies appeared a practical approach for increasing test specificity. We analyzed immune response and testing potential for a spectrum of antigens derived from the spike and nucleocapsid proteins of SARS-CoV-2, developed a dual-antigen testing system in the ELISA format and designed a robust algorithm for data processing. Combining nucleocapsid protein and receptor-binding domain for analysis allowed us to completely eliminate false positive results in the tested cohort (achieving specificity within a 95% confidence interval of 97.2–100%). We also tested samples collected from different households, and demonstrated differences in the immune response of COVID-19 patients and their family members; identifying, in particular, asymptomatic cases showing strong presence of studied antibodies, and cases showing none despite confirmed close contacts with the infected individuals.

## 1. Introduction

COVID-19 is a disease caused by a virus from the *Coronaviridae* family known as SARS-CoV-2. Other members of the *Coronaviridae* family shown to infect humans include four “common cold” seasonal coronaviruses, HCoV-OC43, HCoV-HKU1, HCoV-229E and HCoV-NL63, which cause mild upper and lower respiratory syndromes, as well as SARS (SARS-CoV-1) and MERS (MERS-CoV) coronaviruses [1], which cause severe acute respiratory syndrome and were responsible for outbreaks in 2003 and 2014 in Asia and the Middle East among thousands of people.

SARS-CoV-2 is an enveloped virus; its genome is a single-stranded positive-sense RNA. It was first identified in December 2019 in Wuhan City, China, after several individuals had developed severe pneumonia symptoms resembling SARS-CoV-1 infection [2]. The virus has quickly spread and in March 2020, the WHO officially declared COVID-19 a pandemic. The incubation period of the COVID-19 infection usually ranges from 1 to 14 days. The virus is mainly detected in respiratory secretions, and the general transmission of infection is considered airborne. It has been shown that the virus attaches to the pulmonary cells using their ACE-2 receptors, followed by endocytosis [3]. Immune response is expected to build starting from one week after infection [4].

Serological analysis is based on the identification of antibodies specific to infection agent in serum or other bodily fluids [5]. Serological surveys are used in epidemiological studies to determine the prevalence and spread rate of a disease within a population [6]. The testing also tells whether a specific person has been through a certain infection or not, and thus assess their risks of getting sick and spreading the infection further.

The levels of IgG antibodies for COVID-19 patients were shown to appear starting from a week after the onset of the disease [7] being detectable in most samples after 20–22 days [8]. It has also been shown that asymptomatic individuals tested positive for COVID-19 can develop antibodies, but typically their concentration is lower [9].

The challenge in the antibody analysis is ensuring its sensitivity and specificity [10]. A COVID-19 serological testing system must effectively detect SARS-CoV-2 antibodies in samples and distinguish them from antibodies specific to other infections. Despite availability of a multitude of the newly developed tests, the quality of respective results remains ambiguous in many cases, yielding false positive results. A UC Berkley team COVID-19 Testing Project demonstrated that out of 14 serological tests, only three delivered consistent results, and some cases showed specificity of less than 85% [11], which means one in seven people who were not actually infected obtained a false positive result. Currently, there are over 300 serological tests in development or already available, however, there is growing evidence that while such assays may initially demonstrate good clinical performance regarding sensitivity and specificity on limited sets of samples, diagnostic outcome during large-scale testing in a population may be not as reliable due to, in particular, low titers or cross-reactivity of antibodies [12].

The human body is known to produce multiple types of antibodies in response to a single infection [4]. Testing for antibodies specific to different viral antigens appeared to us a practical approach to increase confidence of the immune response assessment. It was recently shown that a multiple-antigen approach can indeed increase specificity of testing [13]. The focus of this study was to develop an accurate, robust, and reproducible multi-antigen testing approach which would allow for maximum specificity in the ELISA format, while maintaining an adequate sensitivity level. Optimal experiment conditions were set to be determined, and testing capabilities of a spectrum of candidate viral proteins for antigen selection were set to be analyzed.

Most commonly, the serological testing systems use strict cut-offs above which the antibody is considered present. Usually, the data is assumed to follow a normal or half-normal distribution and a cut-off value is set as three standard deviations (SD) above the negative mean distribution [14]. Simple procedure of setting higher cut-offs may increase the specificity of the test, but it will lower its sensitivity. This procedure heavily depends on the results of the analysis of, ideally, a considerable set of negative samples, which are also not expected to yield false positive results; this is not particularly convenient during large-scale testing procedures.

In other approaches involving negative and positive training sample sets, statistical methods, such as the Youden J statistic cutoff, are used to determine absolute antibody threshold concentrations, and in following experiments absolute concentration values of studied samples to said threshold are calculated and compared [13]. However, this also may not be convenient during large-scale analysis, e.g., population screening, where the robustness of the system is crucial and the qualitative tests may show significant bias when switching between laboratories, equipment, and operators.

Here we report development an efficient simple threshold algorithm not involving concentration calculations, which allows for comprehensive interpretation of the ELISA results using single positive controls for each antigen.

## 2. Materials and Methods

All relevant ethical guidelines have been followed; all subjects gave their informed consent for inclusion before they participated in the study. The protocols for obtaining samples were approved by the Ethics committees of respective organizations (Access Biologicals LLC, SDP-001, 12/6/2020; Reprocell USA Inc., C101, 7/7/2020; ProteoGenex, PG-ONC 2003/1, 9/1/2020; from Lenco Diagnostic Laboratory Services de-identified samples were obtained). All necessary patient/participant forms have been signed by participants and archived.

### 2.1. Specimens

The total number of collected samples for this study was 401. From these, 28 serum samples were collected by us throughout the timeline of the study, of which 11 were from COVID-19 patients confirmed by a SARS-CoV-2 RT-PCR assay and others included their household members. We also used 17 negative samples collected before 2019 (pre-pandemic) from different projects of ours. Other specimens were purchased from commercial providers; the complete list is available in Table 1.

### 2.2. Commercial Antigens

Commercial antigens were purchased from GenScript and comprised of the nucleocapsid protein (Z03488), S1 domain of the surface protein (Z03485), RBD, receptor-binding domain of the surface protein (Z03479, Z03483), nucleocapsid and RBD fusion protein (Z03498) and a C-terminal fragment of the nucleocapsid protein, amino acid residues 122–419 (Table 2).

### 2.3. Antigen Production and Purification

The first antigen (RBD) obtained in our laboratory was a fragment of SARS-CoV-2 surface glycoprotein that includes amino acid residues 319–591. It was designed to include the surface glycoprotein receptor-binding domain (amino acid residues 319–541) and 50 additional amino acid residues that contain a strong B-cell epitope, according to the BepiPred-2.0 algorithm [15]. The humanized sequence encoding the 319–591 fragment was fused to a sequence encoding the surface glycoprotein signal peptide (amino acid residues 1–14) and to a sequence encoding a C-terminal hexahistidine affinity tag. The resulting construct was expressed under a CMV promoter in transiently transfected HEK293 cells and purified from the conditioned media on Ni-NTA Sepharose and MEP Hypercel resin. The protein was dialyzed to phosphate buffer saline (pH 7.4) containing 20% glycerol. For the ELISA, the microtiter plate wells were coated with 0.1 µg of RBD per well.

The second antigen (N) was a full-length nucleocapsid protein of SARS-CoV-2. It was cloned into a pET-based vector carrying a C-terminal hexahistidine tag and expressed in a soluble form in a BL21(DE3) *E. coli* strain. Purification from the soluble fraction of the bacterial cell lysate was done on Ni-NTA Sepharose. The resulting protein was dialyzed to 20 mM HEPES-Na, pH7.9, 20% glycerol, 100 mM NaCl, 1 mM DTT, and 1 mM EDTA. For the ELISA, the microtiter plate wells were coated with 0.05 µg of N per well.

We compared antibody detection capabilities between commercial antigens obtained from GenScript and the same antigens custom-produced in our laboratory; the results were consistent and demonstrated no difference regarding false positive and false negative sample rates by any of the algorithms used in this study.

**Figure 1:**
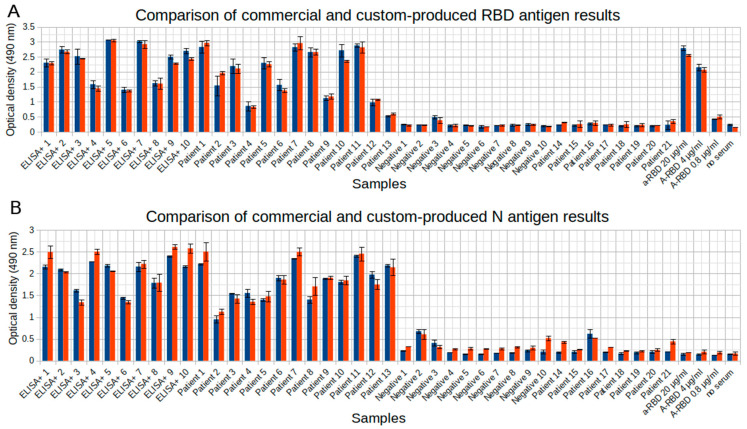
Comparison of commercial (GenScript) antigens (red bars) and antigens produced in our laboratory (blue bars) for the (**A**) RBD antigen and (**B**) N antigen. “ELISA+” samples are confirmed by an alternative ELISA test (ProteoGenex), “Negative” samples are collected before the pandemic, “Patient” samples are collected throughout this study. Experiments were done in two replicates.

An example of comparison of these results is shown in Figure 1, which represents an ELISA experiment data for 10 positive samples confirmed by an alternative ELISA test (ProteoGenex), 10 negative pre-pandemic samples, and 21 patient samples collected by us throughout the study. The ELISA protocol is described below. The plate was processed on a plate reader at 490 nm, and the respective optical density values are shown. The Pearson’s correlation coefficient for the RBD results is 0.994 and for N results is 0.986.

### 2.4. Control Antibodies against RBD and N Antigens

SARS-CoV-2 Spike S1 Antibody (HC2001), Human Chimeric (GenScript A02038) monoclonal anti-RBD antibody was used as the positive control for experiments with human anti-RBD antibodies, and SARS-CoV-2 Nucleocapsid Antibody (HC2003), Human Chimeric (GenScript, A02039) monoclonal anti-N antibody was used as the positive control for experiments with human anti-N antibodies.

### 2.5. Secondary Antibodies

Monoclonal mouse Anti-Human IgG Fc Antibody (clone 50B4A9, GenScript #01854) was used as the secondary antibody. This antibody was purified on Protein A Sepharose.

### 2.6. ELISA Plates Preparation

The SARS-CoV-2 antigens were diluted in 1× PBS and used to coat 96-well ELISA plates (4 HBX, ThermoFisher, Waltham, MA, USA, 3855). The plates were coated with 100 μL of diluted RBD antigen (3.75 μg/mL) or diluted N antigen (4 μg/mL) per well and incubated for 4 h at 20 °C (RT) or overnight at 4 °C. The plates were washed three times with PBS-T (TWEEN 20 at 0.1%, Sigma, St. Louis, MO, USA, P1379) and then blocked with PBS-T + 3% milk powder (weight/volume, “Dry Milk”, Sigma #P4739), at 200 μL blocking solution per well at 20 °C (RT) for 2 h. The blocking solution was removed, and the plates were then dried at 20 °C (RT) for 2 h or overnight. The dried plates were then sealed with MicroAmp™ Clear Adhesive Film (ThermoFisher, 4306311) and stored at 4 °C in a bag with desiccant (Sigma, 1038040001) in Silver Metallized Zipper Pouch Bags (ClearBags, ZBGM4S).

### 2.7. ELISA Protocol

For the ELISA, the wells of the plate were filled with 100 μL of PBS-T containing 1% casein (1× Casein in PBS ready to use solution, ThermoFisher #37528 with 0.1% TWEEN-20 added). The samples were pre-diluted 1:5 with 1× PBS in a PCR plate, then 10 μL was added to the wells. The plate was incubated for 1 h at 20 °C (RT) and washed three times with PBS-T. Anti-human IgG HRP conjugated secondary antibody (Mouse Anti-Human IgG Fc Antibody (HRP) mAb, GenScript, A01854) was diluted 1:3000 in PBS-T containing 1% casein and 100 μL was added to the wells. The plate was incubated for 1 h at 20 °C (RT) and then washed three times with PBS-T. To perform the colorimetric step SigmaFast OPD (Sigma, P9187) was used with a set of 1 gold and 1 silver tablet dissolved in 20 mL of water following manufacturer instructions. 100 μL of SigmaFast OPD substrate solution was added to the wells of the plate and after 10 min incubation the reaction was stopped by addition of 50 μL of 3M HCl. The plate was processed on a plate reader at 490 nm.

### 2.8. Data Processing

For the initial antigen selection procedure, we identified the signal for a negative sample as a false positive if its optical density (OD) value obtained from the reader was above a threshold defined by a commonly used approximation method, where it is set as three standard deviations above the average negative control value [16]. To assess sensitivity during the same initial stage we used a training set of positive samples and indicated the respective result as false negative if the OD value for such sample was less than the highest negative control value, excluding possible false positive results identified in the previous step.

Further in the analysis, for the processing of the developed ELISA dual-antigen system results we designed a specific algorithm described in detail in the Results section.

For comparison of results obtained for different experiment conditions, including using different serum dilutions, different blocking reagents and different antigens, standard deviations and coefficients of variations were obtained, and either null hypothesis significance testing was applied or Pearson’s correlation coefficients were calculated.

To determine the 95% confidence interval (95% CI) for the result assessment regarding the exact binomial Bayesian credible interval (Jeffrey’s interval) [17] method was used.

For the assessment of the efficiency of positive and negative samples separation with two parameters we used a support-vector machines algorithm (SVM) to create linear classifiers [18]. 5-fold cross-validation method was employed to estimate the accuracy of the separation results by splitting the data and computing the prediction score five consecutive times with varying training and testing sets [19,20].

## 3. Results

We analyzed immune response and diagnostic potential for several antigens designed based on the SARS-CoV-2 structure under different experimental conditions. We used serum samples obtained from a general asymptomatic population of pre-epidemic individuals (negative samples) and from patients which tested positive with a SARS-CoV-2 RT-PCR assay or alternative ELISAs (positive samples).

Antigens used for our analysis comprised the full length nucleocapsid protein (N), nucleocapsid protein truncated from the N-terminus (N-truncated), the first domain of the spike protein (S1), the receptor-binding domain of the spike protein (RBD), and the N-RBD fusion construct (N-RBD).

Through a series of experiments, we have obtained optimal conditions for the ELISA experiments, in particular, determining the necessary serum dilution and blocking reagent.

### 3.1. Optimal Serum Dilution

**Figure 2:**
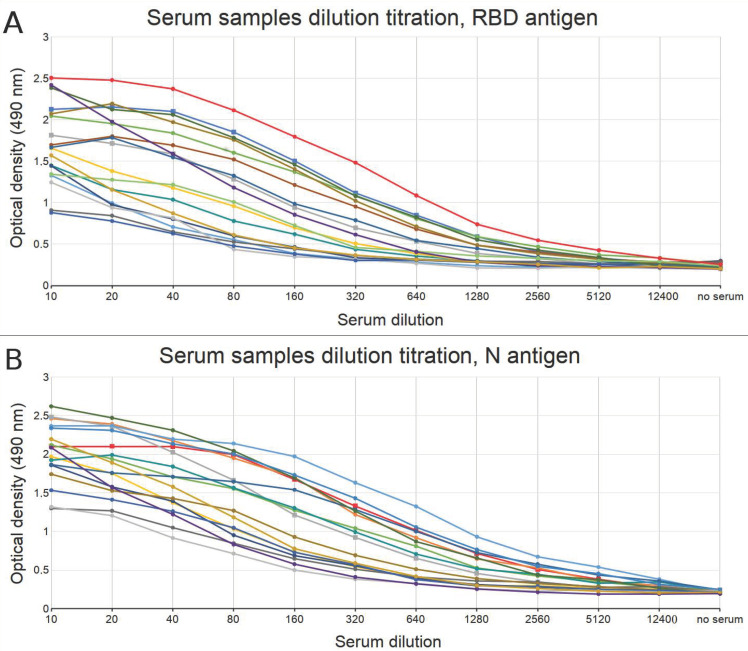
Serum dilution calibration results using (**A**) RBD antigen and (**B**) N antigen for the antibody detection. Red line corresponds to positive antibody control titration results, other lines correspond to COVID-19 positive samples results. Experiments were done in two replicates.

Ten positive serum samples confirmed by an alternative ELISA test (ProteoGenex) and 7 positive serum samples collected by us and confirmed by SARS-CoV-2 RT-PCR assay along with a positive antibody control were used to determine the optimal serum dilution. A series of 2× serum dilutions ranging from 10 to 1240 were prepared and analyzed with ELISA and respective OD signals were obtained on a plate reader at 490 nm. Obtained signals were considered positive if they exceeded the noise level (average value from wells with no serum added) at least two-fold. According to this threshold, all positive samples showed positive signals for dilution up to 80× when RBD antigen was used for the antibody detection, and up to 160× when N antigen was used (Figure 2). For convenience, we have used the dilution of 50× in our further experiments.

### 3.2. Optimal Blocking Reagent

**Figure 3:**
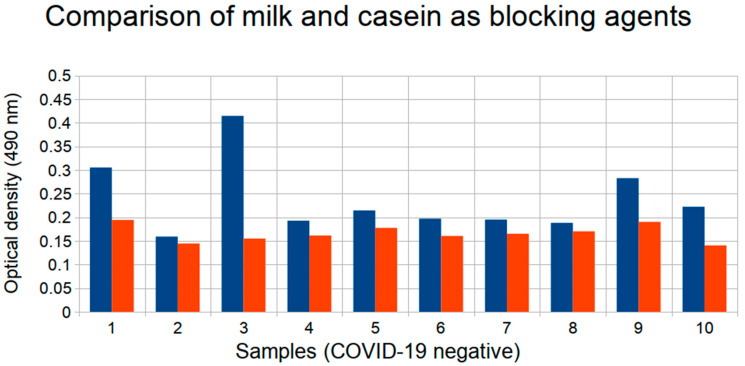
Comparison of milk and casein as blocking agents for ELISA with RBD antigen used for antibody detection on a set of 10 COVID-19 negative samples. Blue bars correspond to results on milk, red bars to casein.

We have tested casein (PBS-T containing 1% casein) and non-fat dry milk (PBS-T containing 1% non-fat dry milk) for the ELISA experiments with 10 positive and 10 negative samples (confirmed by the ProteoGenex ELISA test) with RBD and N antigens. We have calculated the coefficients of variation (CV, the ratio of the standard deviation to the mean value for replicas results) for each sample; for RBD it ranged between of 1.3% and 7.6%, for N it ranged between 0.3% and 9.1%. With casein, the average signal for RBD in the negative samples was lower by 30% (*p*-value 8.8 × 10^−13^) (Figure 3). The average signal of positive samples was not significantly affected; it was higher by 3%, which was comparable with the CV. On the graph it can be observed, that while low-signal samples are usually not greatly affected by the switch of the blocking agent, casein significantly improves the data for negative samples which yielded high signals with milk (see samples 1, 3, 9). We thus concluded that using casein lowers the probability of acquiring false positive results.

For the N-antigen the average signal of negative samples lowered by 14% (*p*-value 0.0013). The average positive signal was likewise not affected (it lowered by 2%, which was comparable with the range of the coefficient of variation).

Since the RBD antigen OD signals were on average lower than those of N, lowering the overall level of negative samples signals and specifically eliminating high signals was an important step in the optimization of the assay. Casein was used as a blocking reagent for further experiments.

The next, most important step, was the selection of antigens.

### 3.3. Antigen Selection

For the initial estimation of immunogenicity of studied antigens, we calculated the crude rate of false positive results using the approach with the threshold of three standard deviations above the average negative control, as described in the Methods. It is worth noting that this threshold rule was very strict, identifying some signals as false positives even when they were lower than all positive sample signals, but during this step our goal was to apply a hard filter to the antigens and select optimal candidates for further analysis on larger datasets. To ensure efficient sensitivity we used six samples from the patients with confirmed COVID-19. No false negative results were obtained for studied antigens at this point.

**Figure 4:**
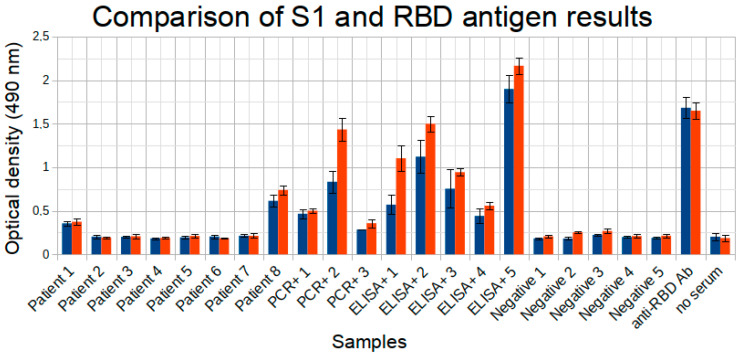
Comparison between S1 (blue bars) and RBD (red bars) antigen results. From left to right: eight results are from patients with uncertain status; three PCR+ and five ELISA+ are COVID-19 confirmed cases; five are negative COVID-19 cases; anti-RBD antibodies used as positive control; no serum (noise level). Experiments were done in two replicates.

We first compared the S1 and RBD antigens. RBD, the receptor-binding domain, is a part of the S1 domain, and they both belong to the HCoV-2 surface protein. The antibody detection results to those two antigens were expected to be similar at least to some degree. To analyze this correlation, we used eight positive samples, eight samples from individuals with an uncertain status (collected from the members of households of the individuals with confirmed COVID-19 diagnosis), and five negative samples. Anti-RBD antibodies were used as positive control. The noise level was assessed in the well where no serum was added (Figure 4).

**Figure 5:**
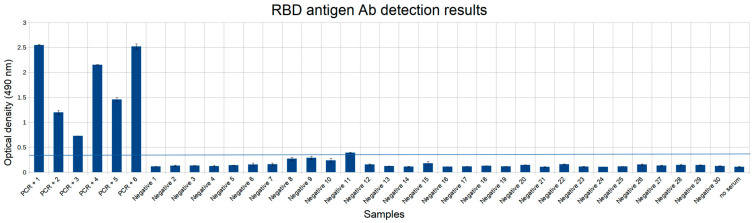
RBD antigen antibody detection results. First 6 samples are positive COVID-19 samples confirmed by PCR (PCR+ 1–6), next 30 samples are negative. Threshold for identifying false positive results is shown in a blue horizontal line. Experiments were done in two replicates.

The S1 antigen demonstrated results highly similar with the RBD antigen results (Pearson’s correlation coefficient 0.96), with RBD overall giving higher signals for all positive samples. For both RBD and S1 all the positive samples yielded values greater than negative samples, so efficient separation between positive and negative samples was possible in both cases, which indicates advantages in testing capabilities for either of these antigens. RBD is a part of the surface protein that was shown to be primary contributor to the neutralizing antibodies development [17]. Out of the two we have thus selected RBD antigen for further analysis. The RBD antigen yielded one false positive result on a larger dataset comprising 30 negative samples, reaching the specificity of 96.7% (95% CI 85.5–99.6%) (Figure 5).

**Figure 6:**
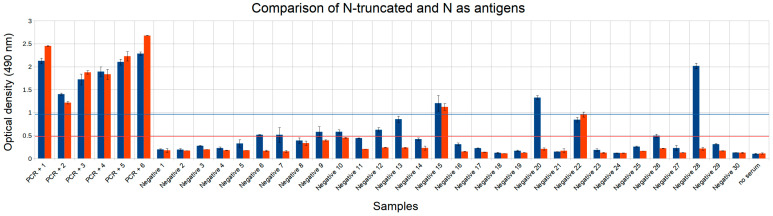
Comparison of N-truncated (blue) and N (red) antigen antibody detection results. The first six samples are positive COVID-19 samples confirmed by PCR (PCR+ 1–6), next 30 samples are negative. Thresholds for identifying false positive results are shown in horizontal lines, blue for N-truncated and red for N. Experiments were done in two replicates.

Next, we analyzed results for the nucleocapsid (N) protein and its truncated version (N-truncated) on the same set of samples. The N-truncated antigen yielded three false positives, demonstrating overall specificity of 90% (95% CI 75.7–97.1%). The full-length N antigen demonstrated two false positive results, corresponding to specificity of 93.3% (95% CI 80.3–98.6%) (Figure 6). Overall N-truncated antigen negative samples values were significantly higher, by 87% on average, indicating larger noise level. N antigen was thus selected for further experiments.

**Figure 7:**
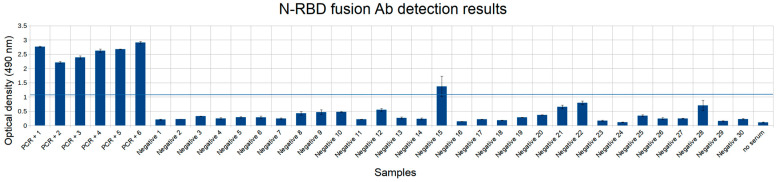
N-RBD fusion antigen antibody detection results. The first six samples are positive COVID-19 samples confirmed by PCR (PCR+ 1–6), next 30 samples are negative. Threshold for identifying false positive results is shown in a blue horizontal line. Experiments were done in two replicates.

Additionally, we analyzed the immunogenicity of the N-RBD fusion construct (Figure 7). Its noise level was comparable to N-truncated antigen, although it yielded only 1 false positive result, similar to the RBD-antigen. Overall, its outcome was promising regarding testing capabilities. The interpretation of the respective results, however, remains a moot point, since it is not possible to determine which viral protein is responsible for binding of the antibodies and the question about their potential neutralizing capabilities could not be easily answered.

The false positive results yielded by the RBD and the N antigens did not overlap. Coming to the conclusion that the combination of N and RBD as two separate antigens might provide maximum specificity of the study, we have developed a testing system in the ELISA format which included both of them (a dual-antigen system). Selected antigen concentrations for the plate coating were 3.75 μg/mL for RBD and 4 μg/mL for N.

The next step was to develop a better threshold algorithm for identifying positive and negative results, assuming the initial false positive filtering method was very strict. In all initial experiments described above, the lowest value among positive samples and the highest value among negative samples differed (lowest positive being higher than highest negative). We considered possible developing an accurate separation procedure to sufficiently distinguish positive and negative samples.

Other steps of the assay development included validating that the false positive results between N and RBD antigens were not shared on other datasets and further testing the system sensitivity.

### 3.4. Positive and Negative Sample Separation with Two Antigens

**Figure 8:**
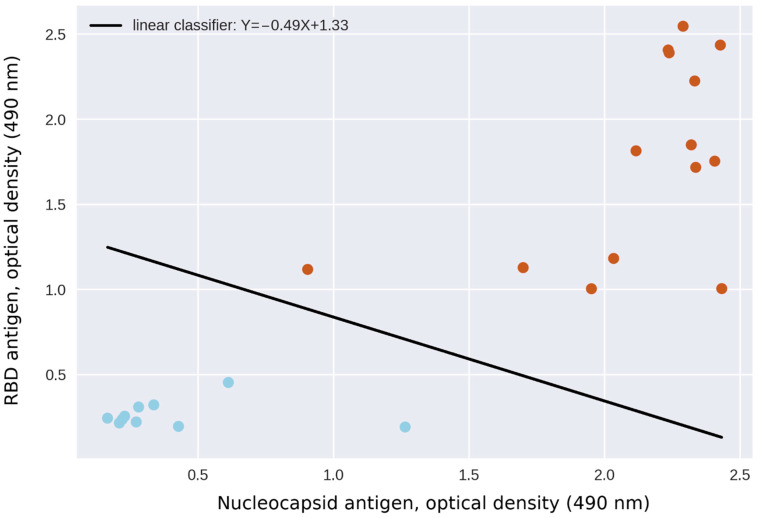
SVM linear formulation for separation of positive (red) and negative (blue) samples. Two parameters defining the 24 samples are RBD and nucleocapsid antigen OD values (*x*- and *y*-axes, respectively).

Using support-vector machines algorithm (SVM) to create linear classifiers with a training set including 24 samples, we have confirmed that the positive and negative samples can be successfully separated using two parameters, namely the OD values of the N antigen and the RBD antigen. The dual-antigen system yielded no false positive and no false negative results (Figure 8).

We used the 5-fold cross-validation method to estimate the accuracy of the separation results as described in the Methods. The results were predicted correctly every time. The F1 score (a weighted average of the precision and recall, which reaches its best value at 1 and worst at 0) was 1 every time.

It is worth noting that the RBD antigen generally provides a wider range of OD results within different positive samples, thus the threshold for separation of positive and negative samples in case of the anti-RBD antibodies must be set quite low. Low thresholds, in turn, may lead to lowering of the specificity, so using two parameters (and, respectively, two thresholds) simultaneously for identification of positive results is justified.

We next performed the same procedure on a larger set of samples, comprising 31 positive samples (confirmed by PCR) and 89 negative samples (120 samples in total). This experiment included three plates, so the OD values were normalized by a positive control of the same concentration on all the plates (anti-N and anti-RBD antibodies).

**Figure 9:**
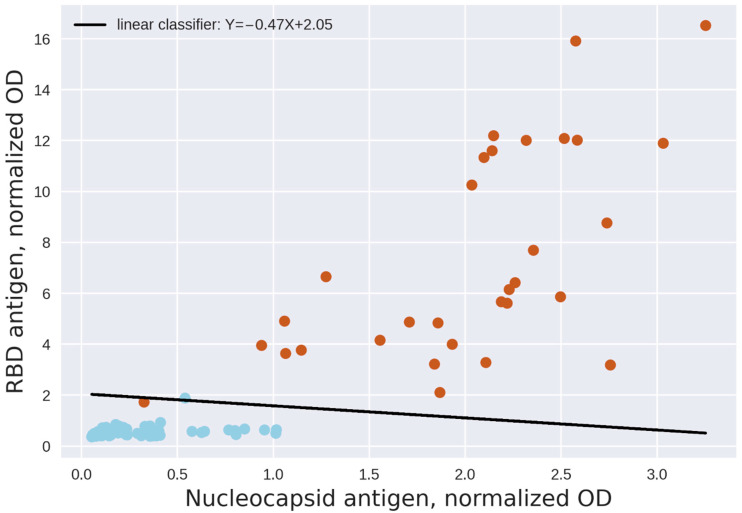
SVM linear formulation for separation of positive (red) and negative (blue) samples. Two parameters defining the 120 samples are OD values normalized by positive control for the N antigen and the RBD antigen (*x*- and *y*-axes, respectively).

Two positive samples had OD values for the N antigen lower than several of the negative sample results (at the same time their RBD values were high). One negative sample, on the other hand, had an RBD value higher than one of the positive samples. While the linear classifier separates most of the samples efficiently, it still yields one false negative result (Figure 9).

The cross-validation method was again used to assess the robustness of this separation, where the prediction score was computed five consecutive times and the results were predicted correctly with F1 scores of 1; 1; 1; 1; 0.91.

These observations demonstrated that positive and negative samples could be efficiently separated with high accuracy on large datasets using two thresholds, yielding only a very small number of false negative results with the right classifier selected. False positive results could be completely avoided by applying a two-parameter threshold system. For a sample to be considered positive, both antigen results should be above a certain value determined for each. We thus developed a respective threshold selection algorithm.

### 3.5. Threshold Algorithm

To be able to separate positive and negative samples during each ELISA experiment with new specimens, we have developed a procedure which uses a single positive control for each antigen as a threshold. The use of positive control allows to identify positive samples without relying solely on an integer number of standard deviations from an average OD value from confirmed negative samples, which requires using training sets for every application of the assay and is inconvenient regarding plate space and sample storage requirements.

For positive control, antibodies against the nucleocapsid protein (anti-N) and the receptor-binding domain of the surface protein (anti-RBD) were used. To determine the correct thresholds for the interpretation of all further ELISA results, we analyzed a training set of negative and positive samples and created a calibration curve for a set of anti-N and anti-RBD antibody dilutions. The concentrations of both positive controls were determined once per their lots and then used for all consequent assay applications.

We select thresholds using the following procedure. Firstly, we determine the bounds for the window which separates positive samples from negative samples (range between values of the highest negative replica and the lowest positive replica). In the rare case where this range is negative, it usually indicates the existence of false positive results; we remove them from the set, defining the result as false positive if its OD value is more than three standard deviations over the average negative sample value.

**Figure 10:**
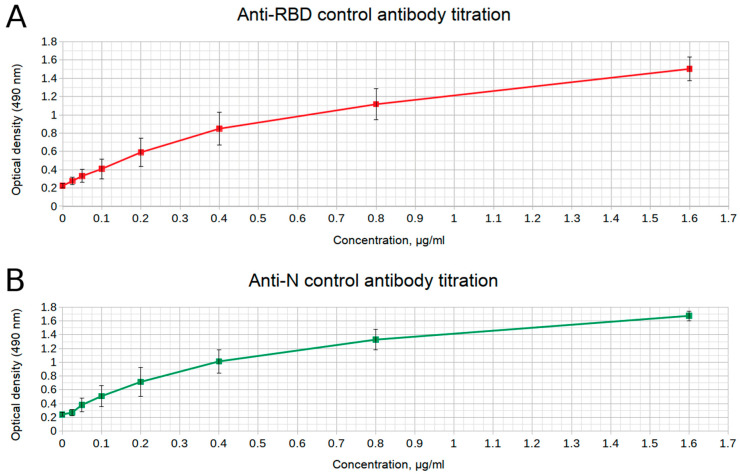
Calibration curves (exemplary) for (**A**) anti-RBD and (**B**) anti-N antibodies. Experiments were done in three replicates.

Secondly, we obtain values from antibody calibration curve (Figure 10) which fall within this window and select the lowest possible value which is at least two-fold higher than average noise level (OD value from the well where no samples or antibodies were added). This is the concentration of the antibody positive control which will be used for further comparison with sample results in all experiments involving the same antibody lot. If a new lot is obtained, the calibration curve is calculated again for validation or optimization.

During further experiments, the OD value of each tested sample is compared to the average OD value of three replicas of the positive control of each anti-N and anti-RBD antibodies. If the sample value is equal or greater than the control, the result for this antigen is considered positive. The result for the sample is considered overall positive if the thresholds for both antigens are positive.

### 3.6. Validation of the Dual-Antigen System

We validated the threshold algorithm on the results obtained from the above-mentioned experiment including 120 samples (31 positive samples confirmed by SARS-CoV-2 RT-PCR assay and 89 negative samples from the pre-pandemic era). The N antigen yielded two false positive results and the RBD antigen yielded one false positive result; they did not intersect. Thus, the threshold algorithm requiring both of these antigens to demonstrate positive results successfully eliminated false positive results, bringing specificity to 100% for the studied cohort (95% CI 97.2–100.0%).

Two false negative results were obtained (both due to N antigen low results), yielding overall sensitivity of the system to 93.6% (95% CI 80.9–98.6%) (Table 3).

The robustness of the dual-antigen system result interpretation was tested with 32 specimens (11 positive and 21 negative) in five repeated experiments done by two different operators over the course of three weeks. Compliant with the threshold algorithm, the sample was indicated as positive if both antigens presented results above respective threshold values obtained using the antibody controls. No false positive results were obtained in either experiment.

Two positive samples had the signal for the N antigen close to the threshold. The first one appeared as false negative in three out of the five experiments and the second appeared as false negative in two out of the five experiments. The ratio to threshold was above 90% in all cases. Results this close to the threshold can be considered ‘borderline’ and such cases may lead to a suggestion for the patient to retake the test in a few days.

### 3.7. Comparison to Another Commercial Test

We have tested samples identified as positive and negative by Siemens CLIA testing system (Lenco Diagnostic Laboratory Services). From 96 samples claimed as negative one gave a positive result with our testing system, and from 96 samples claimed as positive one gave a negative result. Overall, 98.4% results matched between two of the tests (CI 95%, 96.7–99.8%).

### 3.8. Case Studies for the Household Members of COVID-19 Survivors

Samples collected from several households showed a variety of immune responses of family members of COVID-19 patients, who either had or had not reported having symptoms similar to COVID-19. We have tested them using the dual-antigen assay and respective result interpretation algorithm, considering specimen positive if both antigens presented results above respective threshold values obtained using the antibody controls. We have also tested respective samples for other antigens of the study (S1, N-truncated, and N-RBD fusion antigens).

One asymptomatic individual (claiming not to have any kind of manifestation of the disease) demonstrated strong signal for presence of antibodies for both studied antigens, providing a positive result. Their spouse had severe symptoms of the SARS-Cov-2 infection and also had a positive test result. Both of them also tested positive for antibodies with all of the other studied antigens (S1, N-truncated, and N-RBD fusion antigens).

On the other hand, one patient with severe infection symptoms of COVID-19 (confirmed by RT-PCR assay) demonstrated strong signals for N and RBD antigens in the dual-antigen testing system, as well as other studied antigens, but the serum of their live-in partner showed no presence of antibodies to any of the tested antigens.

Another household included six members, all of which claimed to have had mild symptoms of sickness. Three cases were confirmed by an RT-PCR assay to have had SARS-CoV-2 infection and have demonstrated positive results for the dual-antigen assay and presence of antibodies to all other studied antigens. The other three did not show presence of antibodies to any of the antigens from the course of our research.

## 4. Discussion

We developed an assay allowing to comprehensively analyze the immune response to COVID-19, detecting IgG antibodies to viral proteins in serum samples. These types of procedures are essential in epidemiological studies, and also allow to confirm whether a specific person has been previously infected and predicting risks of them spreading the disease further. During the ongoing pandemic of COVID-19 a positive result for a serological test result may indicate whether if it is safe for the person to return to work and re-engage in social activities. However, despite a multitude of emerging tests, the respective data interpretation often remains ambiguous, in particular, yielding a significant amount of false positive results.

We analyzed the testing potential for antigens derived from the surface and nucleocapsid proteins of the SARS-CoV-2 virus, including the whole nucleocapsid protein (N), its C-terminal truncated version (N-truncated), analogs of which had previously showed encouraging outcome in the antibody testing studies with SARS-HCov-1 and OC43 human coronaviruses [21,22], S1 domain of the surface (spike) protein, its receptor-binding domain (RBD), and an N-RBD fusion protein. All antigens showed varying, but promising, results as candidates for the antibody detection.

RBD and S1 demonstrated highly similar results with a low false positive rate. RBD is a part of the surface protein that is responsible for binding to the ACE-2 receptor during infection, so it holds more interest as it shown to be the primary contributor to the neutralizing capabilities of the respective antibodies [23]. Neutralizing antibodies to the causative agent of the disease represent potential prophylactic and therapeutic options and could help guide vaccine design and effect studies. It has been shown in multiple studies both in vitro, with pseudotyped and authentic virus, and in vivo, on models with laboratory animals (including human ACE2 (hACE2)-transgenic mice, adenovirus–hACE2-transduced mice, hamsters, and rhesus macaques), that monoclonal antibodies to RBD demonstrate potent infection-neutralizing capabilities [23,24,25]. In particular, passive transfer of ACE2-blocking monoclonal antibodies as monotherapy protected rhesus macaques from SARS-CoV-2 infection [26]. We thus selected RBD for further experiments as a more interesting research target.

Full-length N antigen showed lower false positive rates than its C-terminal truncated version, and its noise level was significantly lower. The N-RBD fusion noise level was comparable to the N-truncated antigen, but its false positive rate was similar to the RBD-antigen. In case of this fusion, however, it is not possible to determine which viral protein is responsible for binding of the antibodies and the question about assessing their neutralizing capabilities appears more complicated.

Human organism produces polyclonal antibodies specific to multiple viral proteins, so testing simultaneously for multiple antibodies appeared a practical approach for increasing test specificity. Based on our results, we selected the N and the RBD antigens as key components for developing and validating a dual-antigen serological testing system in the ELISA format.

Experiment conditions—such as serum dilution, antigen concentration, and blocking reagent—can be crucial to efficient testing capabilities of the serological assays. Through a series of experiments we obtained optimal experimental conditions for each of the above-mentioned parameters. We have shown that efficient antibody signals could be obtained with serum dilution up to 80× when the RBD antigen was used for antibody detection, and up to 160× with the N antigen. For convenience, we have used the dilution of 50× in our further work. For blocking reagent, casein was selected over non-fat dry milk, since it significantly lowered the signal for negative samples (especially for the RBD antigen), while not affecting the positive sample results. Optimal antigen concentrations for the plate coating were found to be 3.75 μg/mL for RBD and 4 μg/mL for N.

Combining the nucleocapsid and receptor-binding domain of the surface protein for simultaneous analysis allowed us to eliminate false positive results in the experiments, as no samples yielded them simultaneously for both antigens. We consider this to be an important achievement, since incorrectly assuming a person might be safe from the infection due to a false positive serological test result can be particularly dangerous. If one presumes they had already been infected and their immune system had developed protective measures against the virus, they might start to be less careful with pandemic safety measures. This can lead to them getting actually infected and spreading the disease further. Thus, it is crucial to avoid false positive results in testing [27,28].

The robustness of the dual-antigen system result interpretation was tested over the course of three weeks in five repeated experiments done by two different operators and no false positive results were obtained in either experiment. The specificity aspect of the assay can thus be considered robust and reliable. For the N antigen in these experiments, two positive samples demonstrated signal close to the threshold (either slightly above it or slightly below it, the ratio to threshold not exceeding 0.9–1.1). Results this close to the threshold were considered “borderline”, and these cases should be treated as a suggestion for an individual to retake the test in several days, when the immune system may develop a higher level of antibodies. This may happen if the test was taken too early after the patient has been infected.

Testing the system on family members from same households where confirmed COVID-19 patients lived demonstrated several cases of interest. On the one hand, an asymptomatic individual showed strong presence of antibodies to nucleocapsid and surface proteins of the virus. This indicates the importance of serological studies for predictions of the pandemic dynamics, which could not be solely based on data from hospitals. On the other hand, several individuals who have claimed to have COVID-19-like symptoms and were in close contact to confirmed patients did not develop antibodies to either of the studied antigens. This raises questions about the complex nature of immune response to COVID-19, in particular, indicating the need for a more full-spectrum antibody analysis including other viral proteins and peptides, which is planned in the further steps of our study.

In general, we developed and validated a dual-antigen testing system for the analysis of the immune response to COVID-19, which allows to detect IgG antibodies to SARS-CoV-2 proteins in the ELISA format, and designed a robust algorithm for the respective data processing using two single positive controls for each antigen. This allowed us to avoid the more resource-consuming calculations, which could also yield reproducibility difficulties when switching laboratories, equipment, or operators. For studied cohort of samples we achieved 100% specificity and 93.6% sensitivity of the assay, eliminating the false positive results and thus lowering risks of making false assumptions about the patient’s immune response development.

This assay (VirIntel COVID-19 Antibody Test) is currently in clinical use at Clinical Diagnostic Laboratories (CDL), Reston, VA, USA. Further investigations aimed to adapting the Test for dual IgG and IgM detection, and for its use with additional matrices, such as K-EDTA plasma and whole blood from finger prick, are under way. We believe the test and its latter modification, supporting at-home sample collection, will be especially useful in the large-scale serological screenings.

## 5. Patents

Authors are inventors of a pending patent on this work (U.S. Provisional Patent Application, “Dual-Antigen ELISA for Epidemiological and Population Studies of Resistance and Sensitivity to COVID-19”, serial no. 63/088,242, our ref. 6881.143083, Ilya Mazo, Maria Kireeva, Andrei Komarov, Anna Kaznadzey).

## Figures and Tables

**Table 1:** List of serum samples used in the study.

Source	Test Type	Positive	Negative	Unknown
ProteoGenex	ELISA	10	15	
Reprocell USA Inc.	Not applicable		19	
Access Biologicals LLC	PCR	31	89	
Lenco Diagnostic Laboratory Services	ELISA	96	96	
Collected by VirIntel	PCR	11	17	17

**Table 2:** RBD-N fusion protein and N-truncated protein sequences.

RBD-N fusion sequence	SDNGPQNQRNAPRITFGGPSDSTGSNQNGERSGARSKQRRPQGLPNNTASWFTALTQHGKEDLKFPRGQGVPINTNSSPDDQIGYYRRATRRIRGGDGKMKDLSPRWYFYYLGTGPEAGLPYGANKDGIIWVATEGALNTPKDHIGTRNPANNAAIVLQLPQGTTLPKGFYAEGSRGGSQASSRSSSRSRNSSRNSTPGSSRGTSPARMAGNGGDAALALLLLDRLNQLESKMSGKGQQQQGQTVTKKSAAEASKKPRQKRTATKAYNVTQAFGRRGPEQTQGNFGDQELIRQGTDYKHWPQIAQFAPSASAFFGMSRIGMEVTPSGTWLTYTGAIKLDDKDPNFKDQVILLNKHIDAYKTFPPTEPKKDKKKKADETQALPQRQKKQQTVTLLPAADLDDFSKQLQQSMSSADSTQA+linker+SRVQPTESIVRFPNITNLCPFGEVFNATRFASVYAWNRKRISNCVADYSVLYNSASFSTFKCYGVSPTKLNDLCFTNVYADSFVIRGDEVRQIAPGQTGKIADYNYKLPDDFTGCVIAWNSNNLDSKVGGNYNYLYRLFRKSNLKPFERDISTEIYQAGSTPCNGVEGFNCYFPLQSYGFQPTNGVGYQPYRVVVLSFELLHAPATVCGPKKSTNLVKNKCVNF+His tag
N-truncated sequence	LPYGANKDGIIWVATEGALNTPKDHIGTRNPANNAAIVLQLPQGTTLPKGFYAEGSRGGSQASSRSSSRSRNSSRNSTPGSSRGTSPARMAGNGGDAALALLLLDRLNQLESKMSGKGQQQQGQTVTKKSAAEASKKPRQKRTATKAYNVTQAFGRRGPEQTQGNFGDQELIRQGTDYKHWPQIAQFAPSASAFFGMSRIGMEVTPSGTWLTYTGAIKLDDKDPNFKDQVILLNKHIDAYKTFPPTEPKKDKKKKADETQALPQRQKKQQTVTLLPAADLDDFSKQLQQSMSSADSTQA + His tag

**Table 3:** Specificity and sensitivity results for N and RBD antigens separately and combined in the validation test including 120 samples.

Antibody	Performance Measure	Estimate of Performance	95% Confidence Interval
IgG, anti-RBD	Sensitivity	100% (31/31)	(92.3–100%)
IgG, anti-RBD	Specificity	98.9 % (88/89)	(94.9–99.9%)
IgG, anti-N	Sensitivity	93.6% (29/31)	(80.9–98.6%)
IgG, anti-N	Specificity	97.8% (87/89)	(92.3–99.5%)
IgG, Combined	Sensitivity	93.6% (29/31)	(80.9–98.6%)
IgG, Combined	Specificity	100% (89/89)	(97.2–100%)

## Data Availability

All data generated and analyzed during this study are included in this published article. Raw data supporting the findings of this study are available from the corresponding author on request. Data regarding patients presented in this study are not publicly available due to ethical reasons, e.g., containing information that could compromise the privacy of research participants.
